# The Role of Nicotinamide Adenine Dinucleotide Phosphate Oxidases in Lung Architecture Remodeling

**DOI:** 10.3390/antiox6040104

**Published:** 2017-12-19

**Authors:** Anantha Harijith, Viswanathan Natarajan, Panfeng Fu

**Affiliations:** 1 Department of Pediatric, University of Illinois at Chicago, Chicago, IL 60612, USA; harijith@uic.edu; 2 Department of Medicine, University of Illinois at Chicago, Chicago, IL 60612, USA; visnatar@uic.edu; 3 Department of Pharmacology, University of Illinois at Chicago, Chicago, IL 60612, USA

**Keywords:** NADPH oxidase, remodeling, PAH, COPD, asthma, BPD

## Abstract

Chronic lung disorders, such as pulmonary artery hypertension (PAH), chronic obstructive pulmonary disease (COPD), asthma and neonatal bronchopulmonary dysplasia (BPD), are characterized by airway and/or vascular remodeling. Despite differences in the pathology, reactive oxygen species (ROS) have been highlighted as a critical contributor to the initiation and development of airway and vascular remodeling. Nicotinamide adenine dinucleotide phosphate (NADPH) oxidases (Nox) appear to play a pivotal role in lung signaling, leading to marked changes in pulmonary airway and vascular cell phenotypes, including proliferation, hypertrophy and apoptosis. In this review, we summarized the current literature regarding the role of Nox in the airway and vascular remodeling.

## 1. Introduction

The lung tissue consists primarily of airway and vasculature structures. Both structures undergo remodeling under certain disease conditions, such as asthma, chronic obstructive pulmonary disease (COPD), neonatal bronchopulmonary dysplasia (BPD) and pulmonary artery hypertension (PAH) [1,2,3]. Despite differences in the causal agents, these diseases exhibit various degrees of inflammatory changes, airway and vascular structural alterations. Airway and vascular remodeling may be defined as a process of sustained disruption and modification of structural cells and tissues leading to the development of a new airway or vascular structure and consequent new functions. Reactive oxygen species (ROS) are intracellular chemical species that are reactive toward lipids, proteins and DNA. They have been implicated in the pathophysiology of a variety of lung diseases including asthma, COPD, BPD and PAH [4,5,6]. Accumulating evidence has highlighted the importance of Nicotinamide adenine dinucleotide phosphate (NADPH) oxidases in airway and pulmonary vasculature remodeling. The NADPH oxidase family is composed of 7 catalytic subunits termed Nicotinamide adenine dinucleotide phosphate oxidases (Nox) 1–5 and dual oxidase 1 (Duox1) and Duox2, regulatory subunits p22phox, p47phox, Noxo1, p67phox, Noxa1, p40phox and the major binding partner Rac. Nox proteins produce superoxide (O_2_^−^) via a single electron reduction. Superoxide is highly reactive and short lived. Superoxide can dismutate to hydrogen peroxide (H_2_O_2_), spontaneously or enzymatically via superoxide dismutase (SOD). Although production of O_2_^−^ is the main biological function of Nox proteins, much of the signaling that occurs is directly mediated by its dismutation product H_2_O_2_. This is due to the facts that H_2_O_2_ is more stable than O_2_^−^ and is capable of crossing biological membranes. The lung expresses most NADPH oxidases with preferential expression of Nox1 in epithelial and endothelial cells, Nox2 in alveolar macrophages and endothelial cells, Nox4 in smooth muscle cells, fibroblasts and endothelial cells, Duox1/2 in bronchial epithelial cells (reference Table 1). As professional enzymes generating ROS, NADPH oxidases are commonly considered the major source of oxidative stress during acute or chronic inflammation. However, these enzymes have been shown to be involved in a broad range of physiological processes and ROS are increasingly appreciated as critical mediators in a broad range of cellular processes, such as cell proliferation, migration, differentiation, immunomodulation and oxygen sensing [7,8,9,10]. This review will focus on the involvement of NADPH oxidase family in the diseases of asthma, COPD, BPD and PAH with emphasis on pulmonary airway and vascular remodeling.

## 2. Pulmonary Airway and Vascular Remodeling

Lung airway remodeling, a result of chronic and acute inflammation and injury, consists of aspects of airway remodeling and vascular remodeling. Airway remodeling is the results of various cellular and extracellular matrix (ECM) pathological changes, including the changes occurring in airway epithelium, smooth muscle cells, ECM composition and immune cells. Airway epithelia injury and abnormalities in repair are the most likely causes of remodeling [11]. Repeated epithelial injury observed in chronic asthma is associated with release of proinflammatory cytokines and multiple growth factors leading to permanent changes in airway wall morphology [12]. Cigarette smoke is the major cause of COPD. Repetitive exposure of epithelium to this noxious agent leads to marked structural changes to the epithelium with thickening and squamous metaplasia accompanied by elevated mesenchymal responses. Among various growth factors related to airway remodeling, epidermal growth factor (EGF) and transforming growth factor α (TGF-α) play essential roles in airway remodeling. Activation of the EGF receptor promotes both migration and proliferation of epithelial cells [13]. However, EGF signaling is not appropriately activated by the repairing epithelium in asthma [14]. In COPD, cigarette smoke enhanced EGF receptor expression in airways epithelium [15] and elevated expression of both EGF and TGF-β [16]. Thus, the imbalance between proliferative and anti-proliferative signaling represents an important mechanism of airway remodeling.

The pulmonary circulation is a highly specialized vascular bed that physically and functionally connects the heart and the lung. Pulmonary vascular remodeling leads to increased pulmonary vascular resistance and reduced compliance, which play important pathological roles in the development of a variety of pulmonary diseases including COPD, PAH, BPD and asthma [17]. Pulmonary vascular remodeling is the consequence of a wide variety of stimuli exerting on the different components of a blood vessel. Histologically, pulmonary vascular remodeling is characterized by thickening of all three layers of the blood vessel, namely, the adventitia, the media and the intima. Pulmonary vascular remodeling is the result of a wide variety of stimuli, both physical and chemical. These stimuli include mechanical stimuli [18,19], hypoxia [20], growth factors [21,22] and inflammatory cytokines [23]. ROS have been identified to involve all above mentioned stimuli by influencing intracellular signaling pathways activated for vascular remodeling [24,25,26,27]. Pro-inflammatory factors involved in lung remodeling are a major aspect regulated by Nox-derived ROS. It has been shown that lung cells release inflammatory mediators and cytokines/chemokines, such interleukine-1β (IL-1β), interleukine-6 (IL-6), interleukine-8 (IL-8) and tumor necrosis factor-α (TNF-α) in response to oxidative stress through the activation of redox-sensitive transcription factors including hypoxia-inducible factor 1 (HIF-1), nuclear factor-kappa B (NF-κB) [28,29,30,31,32,33].

## 3. NADPH Oxidases and PAH

**Figure 1:**
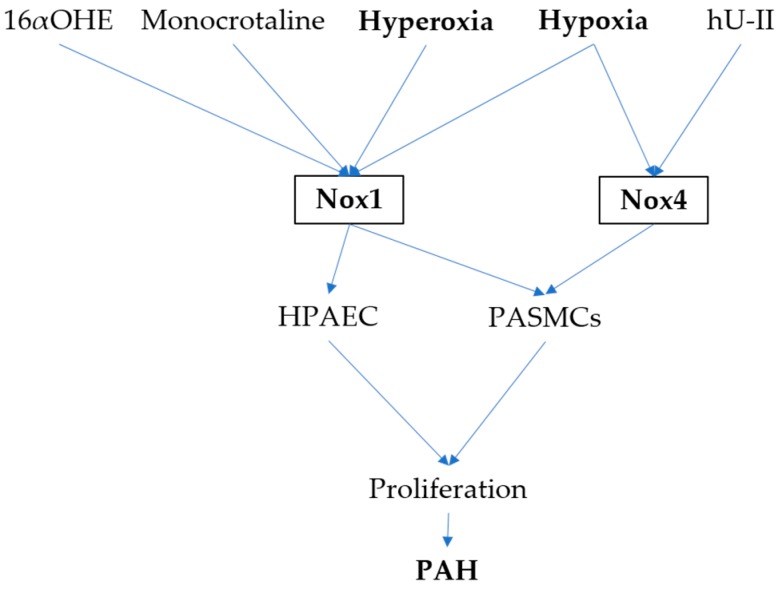
Role of Nox in pulmonary artery hypertension (PAH). Nox1 and Nox4 expressed in human pulmonary artery endothelial cells (HPAECs) and pulmonary artery smooth muscle cells (PASMCs) are activated by various stimuli, among which hyperoxia and hypoxia are the most important ones. reactive oxygen species (ROS) generated by Nicotinamide adenine dinucleotide phosphate oxidases 1 (Nox1) and Nox4 stimulate cell proliferation, which is the major mechanism for PAH.

PAH is characterized by vascular remodeling and upregulated vasoconstriction. Increased pulmonary arterial pressure due to decreased arterial lumen leads to right ventricular failure and death [34]. Accumulating evidence indicates implication of ROS in the pathogenesis of PAH [35] (reference Figure 1). Hyperoxia increased Nox1 expression in mouse lung cell lines and Nox1-deficient mice showed attenuated lung injury induced by hyperoxia [36]. Moreover, Nox1 is increased in neonatal mice with PAH after hyperoxia [37] and in neonatal piglets exposed to hypoxia [38]. Nox1 may be involved in pulmonary vascular remodeling in monocrotaline-treated rats [39]. Consistent to this study, another study showed that monocrotaline induced Nox1 expression and eukaryotic elongation factor 2 kinase (eEF2K) inhibitor, A-484954 attenuated pulmonary artery hypertrophy and fibrosis through inhibition of Nox1 [40]. Nox1 can also be induced by estrogen metabolite, 16α-hydroxyestrone (16αOHE) in human pulmonary artery smooth muscle cells (PASMCs). Estrogen, through estrogen receptor-α, increases Nox1-derived ROS in PASMCs. 16αOHE stimulated transient ROS production and increased Nox1 expression. Nox1^−/−^ but not Nox4^−/−^ mice were protected against PAH and vascular remodeling [41]. One of the early events contributing to the pathophysiology of PAH is endothelial dysfunction [34]. Evidence from endothelial cells support implication of Nox1 in PAH as well. In human pulmonary artery endothelial cells (HPAECs), hypoxia induced Nox1 expression, assembly and oxidase activity leading to elevation in sonic hedgehog and Grem1 expression. Loss of either Nox1, sonic hedgehog or Grem1 attenuates hypoxia-induced HPAECs proliferation [42]. However, there is evidence that is opposite to the findings above. Deficiency of Nox1 leads to reduced number of apoptotic PASMCs, which is implicated in the hypertrophy of pulmonary vessels. Interestingly, deficiency of Nox1 results in a decrease in a voltage-dependent K^+^ channel, Kv1.5 protein and an increase in intracellular potassium levels, indicating a critical role for Nox1 in cellular apoptosis by regulating Kv1.5 and intracellular potassium levels [43].

As the first identified NADPH oxidase, Nox2 is well known for its expression in phagocytic cells as major defense mechanism against bacterial infection, however it is also expressed in cells comprising the vascular wall and is activated by pathways like Nox1. Upon activation, it binds to subunits of p22phox, p47phox, p67phox and Rac1 at the plasma membrane. There is evidence that Nox2 is associated with vasosonstriction and elevated Nox2 correlates with impaired pulmonary vasorelaxation in both lamb and piglet models of neonatal PAH [44,45]. The role of Nox2 in PAH was intensively investigated. It was appreciated that hypoxia-induced PAH was blocked in Nox2 knockout mice [35,46], revealing a pivotal role for Nox2 in the pathogenesis of hypoxic PAH. However, Nox2 mRNA has not been found to be changed in the lung of mice exposed to hypoxia [47]. In contrast, hypoxia induced Nox2 mRNA and protein levels significant upregulation in pulmonary arteries and endothelial progenitor cells of Sprague-Dawley rat concomitant with remodeling in pulmonary arteries and right ventricle [48,49]. In addition, the Nox2 inhibitor apocynin improves oxygenation and decreases ROS in persistent pulmonary hypertension newborn (PPHN) lambs [46].

Originally identified in the kidney, Nox4 is believed to be constitutively active and its regulation is through its expression. Expression levels of Nox4 were reported to be as much as 100-fold greater than other isoforms in vascular smooth muscle cells. Nox4 has been intensively investigated in tumor growth factor (TGF) signaling pathways. TGF upregulates Nox4 expression in various cell types, including PASMCs [50,51,52], airway epithelial cells [53], airway smooth muscle cells [51], artery smooth muscle cells [54] and myofibroblast cells [55]. Meanwhile, TGF plays important role in PAH [56,57,58,59]. In a hypoxia-induced PAH model, Nox4 mRNA was exclusively up-regulated in the lung tissue and Nox4 was identified to be expressed in the media of small pulmonary arteries, with increased labeling intensities after chronic exposure to hypoxia [47]. In PASMCs, Nox4 was localized primarily to the perinuclear space and its expression levels were increased after exposure to hypoxia [47,60]. Hypoxia-induced Nox4 production is through the autocrine pattern of TGF-β1 from PASMCs. In this scenario, exposure of PASMCs to hypoxia leads to TGF-β1 production, which in turn stimulates Nox4 expression in a Smad dependent manner [52]. Hypoxia may also regulate Nox4 expression through NF-Κb [61]. Idiopathic pulmonary arterial hypertension (IPAH) patients have 2.5-fold increase in Nox4 expression, which was localized in the vessel media [47,62]. Human urotensin (hU-II) is a potent vasoactive peptide possibly involved in PAH. It has been shown that hU-II upregulated expression of Nox4 and p22phox in PASMCs and stimulated PASMCs proliferation in a ROS dependent manner and Nox4 activation resulted in mitogen-activated protein kinase p38, extracellular signal-regulated kinases 1/2 (Erk1/2) and protein kinase B (Akt) activation, which subsequently enhanced plasminogen activator inhibitor-1 (PAI-1) expression and increased proliferation of PASMCs [50]. Nox4 may also contribute to PASMCs proliferation through cyclin D1, which regulates the transition from G0/G1 to S phase in the cell cycle, resulting in the activation of genes that are necessary for cell-cycle progression. Cyclin D1 was observed to increase in PPHN lungs and PASMC [46] and PASMCs isolated from monocrotaline-treated rats [39]. Inhibition of Nox4 by small interfering RNA decreased cyclin D1 expression in PPHN PASMC, indicating Nox4 regulates PASMCs proliferation through cyclin D1. These results support an important role for Nox4 in PAH through regulating vascular remodeling.

Nox proteins’ cofactor p47 might also play roles in PAH. P47 has been shown to mediate ROS in human lung microvascular endothelial cells (HLMVECs) in response to hepatic growth factor (HGF) [63]. A recent study showed that hyperoxia induced sphingosine kinase 1 (SphK1)-dependent sphingosine-1-phosphate (S1P) accumulation in HLMVECs. S1P in turn stimulated p47 translocate to cell periphery and ROS generation [64]. In another report, SphK1/S1P axis was shown to promote PASMCs proliferation [65]. Therefore, it is possible that the proliferative effect of SphK1/S1P observed in PASMCs is mediated by p47-dependent ROS.

## 4. NADPH Oxidases and COPD

COPD is characterized by inflammation in small airways and lung parenchyma and resultant airway destruction with progressive, irreversible airflow restriction. The complex pathology of COPD involves distinct cellular responses of different regions of the respiratory tract to stimuli, especially cigarette smoke. Among various factors, TGF, EGF receptor, matrix metalloprotease (MMP) and inflammatory mediators, oxidative stress appears to play important roles in the pathology of COPD. There is clear evidence of oxidative stress in COPD patients compared with non-smoking controls [66,67,68,69]. H_2_O_2_ levels in the exhaled breath condensate of COPD patients were much higher than that of healthy controls. Even the levels of H_2_O_2_ were associated with the severity of the disease [67]. Other biomarkers that represent oxidative stress were identified to be increased in COPD as well. For example, isoprostanes, the oxidized products of arachidonic acid, were found in exhaled breath condensate of COPD patients [68]. Malondialdehyde (MDA), a product of fatty acid peroxidation, was significantly higher in exhaled breath condensate of COPD patients than healthy controls. Meanwhile, serum levels of MDA were found to correlate with COPD severity [70]. ROS can be generated in multiple types of cell, which are implicated in COPD.

In COPD, smooth muscle in the small airways exhibit hyper responsiveness and higher contractility, which contribute to airway remodeling and thickening. Increased airway smooth muscle mass is related to the severity of COPD [71]. Given the fact that oxidative stress and oxidative damage play a pivotal role in the pathogenesis of COPD [72], Nox proteins expressed in airway smooth muscle cells may play important role in COPD. Indeed, airway smooth muscle cells express Nox4 [51,73,74,75], p22 [76] and p67 [77]. As the predominant Nox in airway smooth muscle cells, Nox4 has been shown to be upregulated in airway smooth muscle cells of COPD patients [74] and correlate to the severity of COPD [71].

The airway epithelium is one of the first targets of environmental factors implicated in the pathogenesis of COPD and is likely to have impacts upon COPD progression, while there is little evidence of abnormal ROS production from the epithelium. Nevertheless, few studies still revealed the implication of airway epithelial Nox proteins in COPD. Cigarette smoke induced Nox activity through kinin B1 receptor in human alveolar epithelial cells [78]. Nox1, Duox1, Duox2 and DuoxA2 were upregulated in isolated tracheobronchial epithelial cells from COPD patients [79]. Other study showed that expression of Duox1 in the airway brushed tracheal and bronchial epithelium of smoker was significantly downregulated, whereas Duox2 was upregulated [80]. Although these studies didn’t show many mechanisms of how Nox proteins from airway contribute to the pathology of COPS, they still provided evidence that ROS derived from Nox proteins might represent certain aspects of the pathology of COPD.

COPD is characterized by airway narrowing, which is determined by alteration of extracellular matrix (ECM) protein components [81]. ECM is a protein network mainly composed of compliant elastin fibers, collagens, fibronectin, tenascin and proteoglycans, all secreted and organized by the cells embedded with this matrix. The obvious changes in ECM relevant to COPD are reductions in the expression or functional organization of elastic fibers and proteoglycans in the airways and parenchyma [82,83]. Fibrosis of the small airways with increased expression of tenascin-C, fibronectin and possibly collagens in patients with mild to moderate COPD can be observed. Oxidation of ECM proteins has emerged as important mechanism of ECM alteration relevant to COPD and fibrosis. TGF-β appears to be a prominent factor capable of stimulating ECM production. Like what was discussed in the PAH, many pro-fibrotic effects of TGF-β are dependent on Nox4. Inhibition of Nox4 suppressed TGF-β-induced expression of fibronectin, collagen I, α-smooth muscle actin [84]. Similar observations were noted in fetal lung mesenchymal cells and pulmonary fibroblast cells. ROS can lead to carbohydrate oxidation on glycosaminoglycans yielding α-hydroxyalkyl radicals capable of catalyzing reactions with nearby C–OH and C–OR bonds, resulting in the oxidation of collagens, elastin, fibronectin, laminin and glycosaminoglycans [85]. Oxidation of ECM leads to destabilization of interactions between ECM components and growth factors. Oxidation of perlecan leads to proteolysis of fibroblast growth factor 2 (FGF2), which binds to perlecan via its heparin sulfates. Collagen III is a homotrimer linked each other by three disulfide bridges, which are important for maintaining the folding and stability of the molecule [86]. There is an important auto-antigen recognition mechanism for in collagen V, which is implicated in the pathogenesis of fibrosis [87].

As part of COPD, emphysema is characterized by small airway inflammation and airspace enlargement due to lung alveolar destruction [88]. In human emphysematous lungs, the number of Nox2-positive cells was elevated, whereas increased Nox2 and Nox1 mRNA expression was noted in mouse emphysematous lungs. Nox2 but not Nox1-deficient mice were protective against elastase-induced alveolar enlargement. The site of Nox2 exerted its effects was demonstrated at alveolar macrophage [89]. Compared to non-smokers, COPD patients exhibit higher neutrophil count in both bronchial alveolar lavage and in the sputum [90] and neutrophil from COPD patients appeared to be more effective in producing ROS [91], which is believed to be generated by Nox2. Excessive ROS in leukocytes enhanced inflammation through NF-κB-mediated transcription of inflammatory mediators [92]. Activation of Nox2 in leukocytes was mediated by Mac-1, which is an inegrin molecule expressed in neutrophils and this molecule is involved in neutrophil emigration and recruitment and adhesion of neutrophils to the endothelium. COPD patients exhibit significant upregulation of Mac-1 [91]. Phosphorylation of Mac-1 leads to Nox activation and the crosslinking of Mac-1 induces ROS burst in neutrophil [93,94,95].

Toll like receptors (TLRs) are well known for their ability to recognize microbiology. The infection unrelated roles for TLRs has newly emerged [96,97,98]. TLR4 deficiency mice has been shown to exhibit emphysema as they aged [99]. The underlying mechanism of this observation is that Nox3 was upregulated in the lungs and endothelial cells of TLR4 deficiency mice, resulting in increased oxidant generation and elastolytic activity. However, other Nox proteins may play controversial roles to Nox3. One study revealed that Nox2 and p47 knockout mice were more susceptible to cigarette smoke-induced inflammatory response and airspace enlargement. This was associated with enhanced activation of TLR4-NF-κB pathway [100].

## 5. NADPH Oxidases and Asthma

**Figure 2:**
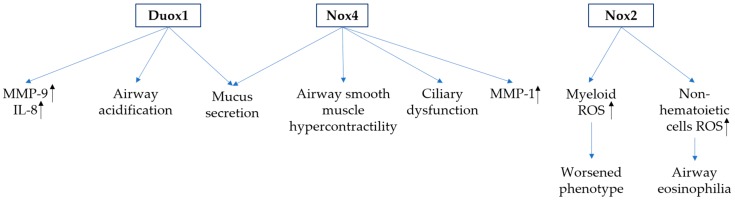
Role of Nox in asthma. Nox2, Nox4 and dual oxidase 1 (Duox1) play critical roles in asthma through various mechanisms. Both Duox1 and Nox4 stimulate mucus secretion and matrix metalloprotease (MMP) production in airway epithelial cells. In addition, Nuox1 enhances airway acidification and Nox4 induces ciliary dysfunction and airway smooth muscle hypercontractility. Nox2 plays distinctive roles in asthma. Nox2 expressed in myeloid and non-hematoietic cells plays distinctive roles in asthma. Nox2 expressed in myeloid cells is believed to mediate the worsened phenotype of asthma, while Nox2 expressed in the lung structure cells mediates airway eosinophilia.

Asthma is a chronic inflammatory disease of the airways, characterized by remodeling of the airway leading to enhanced airway hyper responsiveness and increased mucus secretion and changes in the airway vasculature associated with remodeling of the airway and excessive airway narrowing [101,102]. Airway remodeling of asthma is characterized by subepithelial fibrosis and smooth muscle hyperplasia, which are mediated by cytokines, such as TGF-β [103], EGF [104] and periostin [105]. Accumulating evidence appreciates implication of ROS in the extent of ongoing inflammation and the severity of asthma [106,107,108] (reference Figure 2).

Duox exist as two isoforms, of which Duox1 is primarily expressed in the tracheobronchial epithelium, whereas Duox2 has been detected in salivary or submucosal glands [109,110,111]. In addition to postulated roles in airway host defense, recent studies have suggested alternative functions of airway epithelial Duox1 in mediating asthma. Mucous metaplasia and airway remodeling as hallmarks of allergic asthma have been associated with expression and activation of epidermal growth factor receptor (EGFR) signaling [112]. Constitutive EGFR activation has been associated with cysteine oxidation, which was diminished by pharmacologic or genetic inhibition of the epithelial Duox1 [113]. Targeted inhibition of airway Duox1 in mice reversed symptoms of asthma [113]. Meanwhile, Duox1 regulated mucus secretion through mediating TNF-alpha-converting enzyme (TACE) cleavage into soluble TGF-α, which mediates mucin expression [114]. Other mechanisms of Duox1 regulation of asthma include enhanced inflammatory mediators including IL-8 or matrix metalloprotease-9 (MMP-9) [12,115] and increased airway acidification [116].

In addition to the role of Duox in asthma, Nox4 was also identified to express along with Duox1 and Duox2 in bronchial biopsy specimens by using microarray [75]. Increased Nox4 appeared to associate with airway smooth muscle hypercontractility, which was abrogated by genetic inhibition of Nox4 by small interfering RNA and pharmacological inhibition [75]. Bronchial epithelial ciliary dysfunction was observed in neutrophilic asthma but not in nonneutrophilic asthma. Nox4 and Duox1 were both elevated and Duox2 was elevated in nonneutrophilic asthma. Pharmacologic inhibition of Nox4 improved ciliary function in ex vivo epithelial strips and abolished ciliary abolished ciliary dysfunction in the murine asthma model with no reduction in inflammation [117]. Moreover, Nox4 was identified to contribute to regulating epithelial signaling pathways that promote the production of mucin 5AC (MUC5AC) or matrix metalloprotease (MMP)-1 [118,119]. In normal human nasal epithelial (NHNE) cells Nox4 was the only Nox protein up-regulated by exogenous H_2_O_2_ stimulation. Nox4 was responsible for the increased oxidative stress in NHNE and contributed to the increase in MUC5AC production. Same effects of Nox4 were observed in human lung mucoepidermoid carcinoma cell line NCI-H292 cells as well. Erk kinase seems to mediate Nox4 induction in NHNE cells by exogenous H_2_O_2_. However, Erk kinase may be regulated by Nox4 in alveolar epithelial cell line A549, suggesting a reciprocal regulation mechanism between Nox4 and Erk kinase [118]. Diesel exhaust particles (DEP) induced MMP-1 production in a Nox4-dependent manner. Inhibition of Nox4 abolished DEP-induced ROS production, MMP-1 mRNA expression and Erk phosphorylation [118].

Eosinophils and neutrophils, both of which express Nox2, perhaps the major source of ROS during allergic inflammation. Eosinophils play pivotal roles in the pathophysiology of asthma and exacerbations [120,121]. It is believed that ROS production by these myeloid cells contributes to asthma development. As early responders and regulator of innate and adaptive immune responses during allergic inflammation, eosinophils might also take advantage of Nox2-dependent pro-inflammatory signaling, although this possibility has not yet been tested. On the contrary, Nox2 might negatively regulate acute allergic reactions through the cross-talk between T-lymphocytes and macrophages to limit the inflammatory response [122]. Accordingly, Nox2 deficiency resulted in enhanced recruitment of inflammatory cells to the airways and cytokine production, which worsens the asthmatic phenotype compared with wild type mice [123]. Nevertheless, there is evidence indicating a role for Nox2 within structural cell types rather than inflammatory cells contributes to asthma. This study took advantage of chimera of Nox2-deficiency mice, in which Nox2 was absent only in non-hematopoietic cell types, which showed that only non-hematopoietic Nox2 contributes to development of airways eosinophilia [124].

Of note, in addition to endogenous Nox, exogenous Nox also contributes to asthma. One study showed that pollen contains intrinsic Nox activity. Removal of pollen Nox activity reduced antigen-induced allergic airway inflammation [125].

## 6. NADPH Oxidases in Neonatal BPD

Hyperoxia induced Nox1 is an important contributor to ROS production and disruption of the alveolocapillary barrier during hyperoxia [36]. Nox1 is an upstream actor in oxidative stress-induced acute lung injury involving jun N-terminal kinase (JNK) and Erk1/2 kinase pathways in mice. Increased expression of the reactive oxygen species generating enzyme, Nox1, was noted with hyperoxic exposure in the young but not adult lung [37]. Nox1 appeared to mediate mitochondrial ROS generation in early postnatal hyperoxia-induced phenotype comparable to BPD [126], which is characterized by decreased alveolarization and increased muscularization of resistance pulmonary arteries.

Endothelial activation mediated by lipopolysaccharide (LPS) contributes to lung inflammation and alveolar remodeling seen in premature infants with BPD. Aberrant pro-inflammatory angiogenesis following endothelial dysfunction is noted in chronic inflammatory disorders [127]. It has been shown that Nox2 silencing or use of conditioned media from Nox2-silenced cells attenuated LPS mediated angiogenic responses. This demonstrates the importance of Nox2 in regulating pro-inflammatory Ang2-dependent angiogenesis. Further it has been shown that Nox2 regulates Ang2 and vascular endothelial growth factor-A (VEGF-A) expression in pulmonary endothelial cells through the IKKβ/NF-κB and MAPK/AP-1 pathways [128,129]. Thus, inhibiting Ang2 using antibodies or modulating Nox2 activity could emerge as therapeutic strategies to decrease lung injury in bacterial sepsis. Studies that inhibit Nox (VAS2870) or suppress superoxide (PEG-SOD) suggest that superoxide contributes to LPS mediated induction of angiogenic markers. Data from studies support a role for Nox2-induced ROS in LPS-mediated Ang2 and VEGF-A expression.

Our data have also shown that sphingosine 1 phosphate (S1P) plays a significant role in the pathogenesis of BPD by acting through Nox2 and Nox4 [130]. Sphk1^−/−^ neonatal mice exposed to hyperoxia showed protection against alveolar simplification compared to wild type and Sphk2^−/−^ hyperoxic controls. This was accompanied by a reduction in expression of Nox2 and Nox4. In another experiment, we noted that Sphk1^−/−^ juvenile mice exposed to hyperoxia showed reduced concentration of H_2_O_2_ in bronchial alveolar lavage (BAL) suggesting reduced production of ROS in lungs [64]. In vitro experiments using HLMVECs showed that S1P activated Nox2 by activating the p47 component. This was demonstrated under hyperoxia accompanied by increased production of S1P. P47 activation and ensuing ROS production was inhibited by SphK1 inhibitor PF-543. We further demonstrated that S1P acts through S1P receptor 1 or 2 to activate p47phox followed by production of ROS. The role of p47 in Nox2 activation was supported by a recent study [131], in which p47 was found to be phosphorylated in invariant natural killer T (iNKT) cells underwent hypoxia-reoxygenation. iNKT cells isolated from p47^−/−^ mice had impaired ROS generation, which is believed to be mediated by Nox2.

## 7. Conclusions

With increasing recognition of ROS as critical factors in the pathogenesis of various diseases, Nox inhibitors are the most promising therapeutic option for diseases associated with oxidative stress including lung diseases [132]. Both airway and vascular remodeling are a complex, multi-factorial processes, influenced by multiple physical and chemical stimuli. Undoubtedly, they have their own unique mechanisms but they do share some common mechanisms. For example, both vascular vessels and airway contain smooth muscle cells, which from both tissues are all sensitive to TGF stimulation and play critical roles in remodeling. In view of the complexity of remodeling, targeting Nox proteins-derived ROS might represent a promising strategy for treatment of remodeling-related lung diseases. Targeting Nox1/4 with a pharmacological inhibitor, GKT137831, attenuated hypoxia-induced pulmonary smooth muscle cell proliferation, vascular remodeling and the development of PAH [133]. The Nox inhibitor and ROS scavenger, apocynin, has been shown to reverse the hypoxia-induced decrease in Kv current density related to intracellular Ca^2+^ concentration and associated smooth muscle cells contraction [134] and airway inflammation [135]. N-acetylcysteine (NAC) as a precursor of grutathione (GSH) synthesis has been shown to reduce cigarette smoke-induced abnormalities in alveolar macrophages, fibroblast and epithelial cells [136,137,138,139]. Treatment with NAC in humans increased lung lavage GSH levels, decreased bronchial alveolar lavage polymorphonuclear leukocyte [140]. Administration of NAC in COPD patients reduced sputum eosinophilic cation protein concentrations and the adhesion of polymorphonuclear leukocytes [141]. Pharmacologic inhibitor against Nox4 appears to be effective as an anti-fibrotic agent in preclinical models [142]. Diverse expression of various Nox isoforms in different cell types within the bronchi, lung parenchymal cells and immune cells adds to the great complexity with respect to contributions of ROS to various lung disorders. However, our understanding of the pathophysiological roles of Nox family in the lung is still in its early stage. Advances in understanding the mechanisms of Nox family in disease pathogenesis will expedite the eventual development and test of Nox inhibitors in specific lung diseases. As Nox family has broad pathophysiological roles in different cell types within the lung, defining their pathological context is critical in designing informative preclinical and successful clinical studies. Avenues such as validation of the expression and localization of Nox isoforms within the lungs and identifying Nox proteins as biomarkers of disease progression deserve further studies.

## Figures and Tables

**Table 1:** Lung tissue distribution of Nox proteins.

Type of Nox	Cell Type Where Found
Nox1	Smooth muscle, endothelium, upper airway epithelium
Nox2	Inflammatory cells (macrophage and neutrophils), mesenchymal cells, smooth muscle, endothelium, upper and lower airway epithelium
Nox3	Inducible in lung endothelium
Nox4	Inflammatory cells (macrophage and neutrophils, mesenchymal cells, smooth muscle, endothelium, lower airway epithelial cells
Nox5	Smooth muscle, endothelium
Duox1	Upper airway epithelium
Duox2	Upper airway epithelium

Nox: Nicotinamide adenine dinucleotide phosphate oxidases; Duox: Dual oxidase.
